# Real-Time Hand Gesture Recognition Using Surface Electromyography and Machine Learning: A Systematic Literature Review

**DOI:** 10.3390/s20092467

**Published:** 2020-04-27

**Authors:** Andrés Jaramillo-Yánez, Marco E. Benalcázar, Elisa Mena-Maldonado

**Affiliations:** 1 Artificial Intelligence and Computer Vision Research Lab, Department of Informatics and Computer Science, Escuela Politécnica Nacional, Quito 170517, Ecuador; marco.benalcazar@epn.edu.ec (M.E.B.);; 2 School of Science, Royal Melbourne Institute of Technology (RMIT), Melbourne 3000, Australia

**Keywords:** human–computer interaction, hand gesture recognition, systematic literature review, machine learning, electromyography, real-time systems

## Abstract

Today, daily life is composed of many computing systems, therefore interacting with them in a natural way makes the communication process more comfortable. Human–Computer Interaction (HCI) has been developed to overcome the communication barriers between humans and computers. One form of HCI is Hand Gesture Recognition (HGR), which predicts the class and the instant of execution of a given movement of the hand. One possible input for these models is surface electromyography (EMG), which records the electrical activity of skeletal muscles. EMG signals contain information about the intention of movement generated by the human brain. This systematic literature review analyses the state-of-the-art of real-time hand gesture recognition models using EMG data and machine learning. We selected and assessed 65 primary studies following the Kitchenham methodology. Based on a common structure of machine learning-based systems, we analyzed the structure of the proposed models and standardized concepts in regard to the types of models, data acquisition, segmentation, preprocessing, feature extraction, classification, postprocessing, real-time processing, types of gestures, and evaluation metrics. Finally, we also identified trends and gaps that could open new directions of work for future research in the area of gesture recognition using EMG.

## 1. Introduction

The increase in computing power has brought the presence of many computing devices in the daily life of human beings. A broad spectrum of applications and interfaces have been developed so that humans can interact with them. The interaction with these systems is easier when they tend to be performed in a natural way (i.e., just as humans interact with each other using voice or gestures). Hand Gesture Recognition (HGR) is a significant element of Human–Computer Interaction (HCI), which studies computer technology designed to interpret commands given by humans.

HGR models are human–computer systems that determine what gesture was performed and when a person performed the gesture. Currently, these systems are used, for example, in several applications, such as intelligent prostheses [1,2,3], sign language recognition [4,5], rehabilitation devices [6,7], and device control [8].

HGR models acquire data using, for example, gloves [9], vision sensors [10], inertial measurement units (IMUs) [11], surface electromyography sensors, and combinations of sensors, such as surface electromyography sensors and IMUs [12]. Although there are different options for data acquisition, all of these options have their limitations; for example, gloves and vision sensors cannot be used by amputees; gloves can constrain normal movement, especially in cases involving the manipulation of objects; vision sensors can have occlusion problems, and changes of illumination and changes in the distance between the hands and the sensors; and IMUs and surface electromyography sensors generate noisy data [13,14]. Even though all these devices collect data related to the execution of a hand movement, surface electromyography sensors also extract the intention of the movement. This means that these sensors can also be used with amputees, who cannot execute the movements, but have the intention to do so [15,16].

Surface electromyography, which we will refer to from now on as EMG, is a technique that records the electrical activity of skeletal muscles with surface sensors. This electrical activity is produced from two states of a skeletal muscle. The first state is when a skeletal muscle is at rest, where each of the muscular cells (i.e., muscle fibers) has an electric potential of approximately –80 mV [15]. The second state is when a skeletal muscle is contracted to produce the electric potential that occurs in a motor unit (MU), which is composed of muscle fibers and a motor neuron. These electric potential differences are produced when a motor neuron activates a neuromuscular junction by sending two intracellular action potentials in opposite directions. Then, they are propagated by depolarizing and re-polarizing each one of the muscle fibers [16]. The sum of the intracellular action potentials of all muscle fibers of a motor unit is called a motor unit action potential (MUAP). Therefore, when a skeletal muscle is contracted, the EMG is a linear summation between several trains of MUAPs [15].

There are two types of muscle contractions: static and dynamic. In a static contraction, the lengths of the muscle fibers do not change, and the joints are not in motion, but the muscle fibers still contract, for example, when someone holds his/her hand still or to make the peace sign. While in a dynamic contraction, there are changes in the lengths of the muscle fibers, and the joints are in motion, for example, when someone waves their hand to do the hello gesture [17].

The EMG signals can be modeled as a stochastic process that depends on the two types of contraction described above. First, the mathematical model for a static contraction (MMSC) is a stationary process because the mean and covariance remain approximately the same over time, and the EMG depends solely on muscle force [18]. Consider (1):

(1) $$EMG\left(t\right)=\sum_{i=1}^{N}s_{i}\left(t\right)*m_{i}\left(t\right),$$

where *N* is the number of active MUs, $s_{i}\left(t\right)$ is the train of impulses that indicate the active moments of each MU, $m_{i}\left(t\right)$ are the MUAPs of each MU, and * denotes convolution. However, the MMSC can be viewed as a non-stationary process when factors, such as muscular fatigue and temperature affect the EMG [19].

Second, the mathematical model for a dynamic contraction (MMDC) is a non-stationary process, and its mathematical model is similar to the amplitude’s modulation (AM modulation):

(2) EMG(t)=a(t)w(t)+n(t),

where a(t) is a function that indicates the intensity of the EMG signal (i.e., information signal), w(t) is a unit-variance Gaussian process representing the stochastic aspect of the EMG (i.e., carrier signal), and n(t) is the noise from the sensors and biological signal artifacts [17,20].

The mathematical models of EMG are not used in HGR due to the difficulty of parameter estimation in non-stationary processes. However, machine learning (ML) methods are widely used because ML can infer a solution for non-stationary processes [21] using several techniques; for example, covariate shift techniques [21,22], class-balance change [22], and segmentation in short stationary intervals [23].

HGR using ML is just one approach to myoelectric control [24], which uses EMG signals to extract control signals to command external devices [25,26], for example, prostheses [1], drones [8], input devices for a computer [27], etc. There are other approaches that include conventional amplitude-based control, and the direct extraction of neural code from EMG signals. In conventional amplitude-based control, one EMG channel controls one function of a device (e.g., hand open is assigned to one channel, and hand closed to a second channel). When the amplitude of this EMG exceeds a predefined threshold, this function is activated [28,29,30,31]. The direct extraction of neural code from EMGs is another approach, in which the motor neuron spike trains are decoded from EMG signals to translate into commands [32,33,34,35].

For many applications, HGR models are required to work in real time. A human–computer system works in real time when a user performs an action over the system, and this system gives him/her a response fast enough that it is perceived as instantaneous [25]. Moreover, the response time in a real-time human–computer system is relative to its application and user perception [36]. For this reason, the controller delay, which is the response time of an HGR model, has been widely researched. For instance, a user does not perceive any delay when the controller delay is less than 100 ms in the control of devices, such as a key or a switch [36,37]. In HGR using EMGs, Hudgins & Parker et al. [38] stated that the acceptable computational complexity is limited by the controller delay of the system, which must be kept below 300 ms to reduce the user-perceived lag. This optimal controller delay was generally agreed upon by many researchers [39,40]. However, there have been several optimal controller delays reported in the scientific literature, namely 500 ms [41], and 100–125 ms [42] using a box and blocks test, which is a target achievement test.

Most of the real-time HGR models are evaluated using metrics for machine learning, such as accuracy, recall, precision, F-score, $R^{2}$ error, etc. However, this evaluation fails to reflect the performance exhibited in online scenarios as it does not account for the adaptation of users to non-stationary signal features [43,44,45,46,47]. For example, Hargrove et al. [48] demonstrated that the inclusion of transient contractions (i.e., non-stationary signals) in the training data decreases the accuracy, but improves the user performance in a real-time virtual clothespin task. Therefore, in order to evaluate the real-life performance, the real-time HGR models can be evaluated using target achievement tests, such as the box and blocks test [42,49], target achievement control test [50], and Fitts’ law test [51], which is an international standard in HCI (ISO9341-9).

Currently, there are many primary studies regarding real-time HGR models using EMG and ML, which, in several cases, do not have standardized concepts, such as types of models, real-time processing, types of hand gestures, and evaluation metrics. This standardized knowledge is essential for reproducibility and requires a Systematic Literature Review (SLR) of the current primary studies. To the best of our knowledge, there is no SLR regarding these HGR models. Therefore, we developed this SLR to present the state-of-the-art of the real-time HGR models using EMG and ML. Based on this SLR, we make three contributions to the field of HCI. First, we define a standard structure of real-time HGR models. Second, we standardize concepts, such as the types of models, data acquisition, segmentation, preprocessing, feature extraction, classification, postprocessing, real-time processing, types of gestures recognized, and evaluation metrics. Finally, we discuss future work based on the research gaps we identified.

Following this introduction, the article is organized as follows: in Section 2 we describe the methodology used to execute this SLR; in Section 3 we outline the results and the discussion of the data extracted from the primary studies; and Section 4 and Section 5 contain the conclusions and future work respectively.

## 2. Methodology

We developed an SLR based on the methodology proposed in [52,53], which is comprised of five stages: Research Questions (RQs), Search of Primary Studies, Analysis of Primary Studies, Data Extraction, and Threats to Validity.

### 2.1. Research Questions

In this stage, we define the following four research questions according to the research goal, which is to investigate the state-of-the-art of real-time HGR models that use EMG and ML:

- RQ1. What is the structure of real-time HGR models that use EMG and ML?
- RQ2. What is the controller delay and hardware used by real-time HGR models that use EMG and ML?
- RQ3. What is the number and type of gestures recognized by real-time HGR models that use EMG and ML?
- RQ4. What are the results and metrics used to evaluate the real-time HGR models that use EMG and ML?

### 2.2. Search of Primary Studies

In this stage, we search for the primary studies that can answer the four RQs stated in the previous section. This stage has three parts, which were done manually. In the first part, we selected the literature repositories. In the second part, we extracted the keywords of the RQs, and we developed the search strings using these keywords. Finally, we searched the primary studies in the literature repositories using the search strings.

We used four literature repositories: IEEE Xplore, ACM Digital Library, Science Direct, and Springer. We chose these repositories as they have the most primary studies on real-time HGR models that use EMG and ML and also because these repositories have peer-reviewed papers.

The extracted keywords from the RQs (see Section 2.1) are electromyography, hand gesture recognition, real-time, box and blocks, target achievement control, and Fitts’ law. We, then, added the acronym of electromyography (i.e., “EMG”), and real-time variations: online, real time, on line, and on-line. Therefore, the 11 keywords used in this SLR are electromyography, EMG, hand gesture recognition, real time, real-time, online, on line, on-line, box and blocks, target achievement control, and Fitts’ law. Table 1 shows the 16 Search Strings (SS), which were developed with the combination of these 11 keywords and the Boolean operator “AND”. We do not use the keyword myoelectric control because this SLR is focused on HGR using EMG and ML, which is just one segment of the approaches to myoelectric control (see Section 1).

We looked for the published primary studies from 1 January 2013 to 31 December 2019 (i.e., the last day of search in the literature repositories) using the 16 search strings shown in the Table 1. Table 2 shows the 1485 primary studies, which were found in the four literature repositories, IEEE Xplore: 397, ACM Digital Library: 400, Science Direct: 329, and Springer: 359.

**Figure 1:**
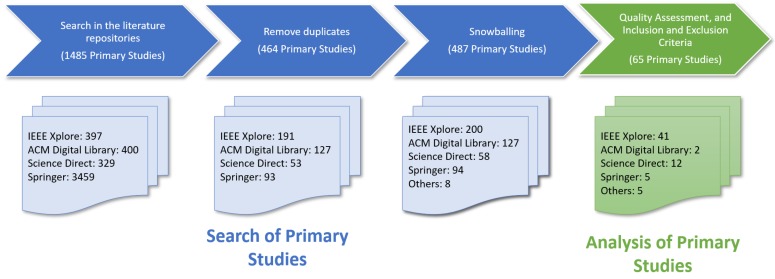
The resulting primary studies after each action carried out in the two stages: search of primary studies and analysis of primary studies.

We discarded 1021 duplicated primary studies of the 1485 primary studies (IEEE Xplore: 206, ACM Digital Library: 273, Science Direct: 276, and Springer: 266). Additionally, we added 23 primary studies to this SLR using the snowballing techniques, which identify the articles that have cited the primary studies found in the literature repositories (i.e., forward snowballing), and the articles from their references (i.e., backward snowballing) [54] (see Table 2). Therefore, we obtained 487 primary studies in total. Figure 1 shows the resulting primary studies after each action carried out in the two stages: the search of primary studies and the analysis of primary studies.

### 2.3. Analysis of Primary Studies

We filtered the 487 primary studies based on the analysis of the titles, abstracts, and conclusions using the inclusion and exclusion criteria, and the assessment questions (see Figure 1). We finally selected 65 primary studies (see Table 3), which were used to answer the four RQs (see Section 2.1).

#### 2.3.1. Inclusion and Exclusion Criteria

We established the Inclusion and Exclusion Criteria based on the RQs (see Section 2.1). These criteria were used to determine if a primary study contributes to answering the RQs. The Table 4 shows the inclusion and exclusion criteria.

#### 2.3.2. Quality Assessment

We defined three assessment questions to evaluate the comprehensiveness, reliability, and applicability of the primary studies. For each question, we established three possible answers with their scores: “Yes” = 1, “Partly” = 0.5, and “No” = 0. Thus, a primary study was rejected if the mean of the three answers is less than 2. The three assessment questions are:

- Were the research objectives of the primary studies clear?
- Was the contribution of the primary study clear?
- Was the structure of the HGR model shown?

### 2.4. Data Extraction

We extracted the data shown in Table 5 from the 65 selected primary studies (SPS), shown in Table 3. This extraction was performed in order to answer the four RQs (see Section 2.1).

### 2.5. Threats to Validity

We discuss the following possible threats to the validity of this SLR and the mitigation of these threats: an incomplete selection of the SPS, inaccurate data extraction, and biased quality assessment.

#### 2.5.1. Incomplete Selection of the SPS

There is a possibility that relevant studies have been omitted for two reasons. The literature repositories may not have had all relevant studies for the four RQs, and the search strings may not have been appropriate for the four RQs. However, the authors performed the following three actions to mitigate these two threats: (1) We developed this SLR based on the Kitchenham methodology [52,53], which was shown in Section 2. (2) In this SLR, the four literature repositories and the ten search strings were proposed by the first author, and the second and third authors assessed the relevance of these literature repositories and search strings. The four literature repositories were assessed in accordance with the criterion that these repositories are the most used in the ML area. The ten search strings were assessed based on the criterion that the keywords and the structures of the search strings are relevant to the four RQs. (3) We applied the snowballing techniques [54] to add 14 SPS to the SLR. This task was performed by the first author, and the third author assessed the relevance of these 14 SPS.

#### 2.5.2. Biased Analysis of Primary Studies

The analysis of the primary studies (see Section 2.3) can be biased for two reasons. The inclusion and exclusion criteria may not be relevant to the four RQs, and the SPS may not be comprehensive, reliable, and applicable. To mitigate these two threats, the authors performed the following two actions: (1) The authors developed formal inclusion and exclusion criteria (see Section 2.3.1) and quality assessment criteria (see Section 2.3.2). These criteria were proposed by the first author, and they were assessed by the second and third authors. (2) The first author selected 65 primary studies reading the title, abstract, and conclusions. However, the first author also read the whole study when the title, abstract, and conclusions were not clear. Furthermore, these 65 SPS were assessed by the second and third authors.

#### 2.5.3. Inaccurate Data Extraction

Generally, the data extracted can be inaccurate for two possible problems: unsystematic data extraction, and the data not being relevant to the RQs. To solve these problems, we extracted the data using a systematic methodology based on the four RQs (see Section 2.4). Moreover, the authors made sure that the extracted data answer the four RQs.

## 3. Results and Discussion

The data extracted from the 65 SPS (see Table 3) are presented and analyzed in five subsections: the study overview subsection and the other four subsections, one per each RQ (see Section 2.1). Although some SPS presented more than one HGR model, we selected the models with the best performance in the evaluation; therefore, we used 65 HGR models for this review.

### 3.1. Study Overview

**Figure 2:**
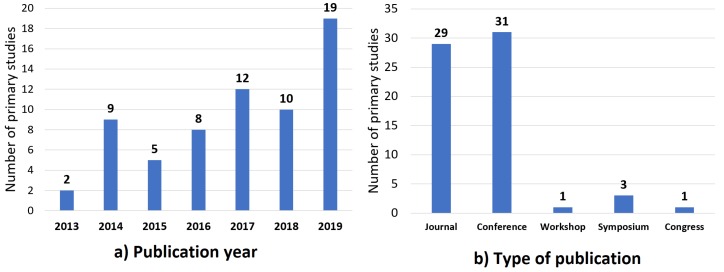
Number of the SPS published per (**a**) year and per (**b**) type of publication.

The study overview shows a general vision of the settings used in the SPS. Among other data, we decided to extract the publication year and the type of publication. Figure 2a shows the number of SPS per year, which has increased steadily since 2013. Moreover, in Figure 2b, we show that most of the SPS were presented in conferences, also see Table 3.

### 3.2. Results of the RQ1 (What Is the Structure of Real-Time HGR Models Using EMG and ML?)

**Figure 3:**
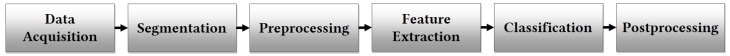
The six stages of the standard structure of the SPS.

We found that the structures of the 65 real-time HGR models are not regular across the studies. However, they have some stages in common, such as Data Acquisition (DA), Segmentation (SEGM), Preprocessing (PREP), Feature Extraction (FE), Classification (CL), and Postprocessing (POSTP). We present a standard structure, considering the frequent stages after they were assembled, the result is illustrated in Figure 3. Note that there are SPS that did not use all stages of the standard structure because Segmentation, Preprocessing, Feature Extraction, and Postprocessing are optional stages (i.e., without them a model is still feasible). Table 6 shows the stages of the standard structure used by the SPS.

Aside from the structure of the models, we identified two types of models: the individual models and the general models. Individual models are trained relying on the gestures (data) of a person and recognize the gestures of that same person. General models are trained with the data of several people and recognize the gestures of any person. We found 44 SPS that developed individual models (SPS 1, SPS 2, SPS 3, SPS 5, SPS 6, SPS 8, SPS 9, SPS 10, SPS 13, SPS 15, SPS 16, SPS 24, SPS 25, SPS 27, SPS 28, SPS 30, SPS 33, SPS 34, SPS 36, SPS 37, SPS 38, SPS 39, SPS 41, SPS 42, SPS 43, SPS 44, SPS 45, SPS 47, SPS 48, SPS 49, SPS 51, SPS 52, SPS 53, SPS 55, SPS 56, SPS 57, SPS 58, SPS 59, SPS 60, SPS 61, SPS 62, SPS 63, SPS 64, and SPS 65), and 11 SPS that developed general models (SPS 7, SPS 11, SPS 17, SPS 22, SPS 23, SPS 26, SPS 31, SPS 32, SPS 35, SPS 40, and SPS 46). The 10 remaining studies do not indicate any type of HGR model. Out of the 11 general models, SPS 35 is the only general model that was evaluated using EMG data from people who did not participate in the training phase. The other 10 general models only used EMG data from people who participated in the training; therefore, it is not possible to conclude that these 10 models are able to recognize gestures of any person.

#### 3.2.1. Data Acquisition

In the Data Acquisition stage, EMGs are acquired from EMG sensors, which can be part of homemade or commercial devices. Table 7 shows the number of sensors, the sampling rates, and the acquisition devices used in the HGR models. We found that 27 HGR models used eight sensors, 21 of them (SPS 2, SPS 3, SPS 4, SPS 7, SPS 8, SPS 9, SPS 13, SPS 17, SPS 18, SPS 19, SPS 20, SPS 34, SPS 35, SPS 36, SPS 40, SPS 44, SPS 46, SPS 47, SPS 52, SPS 56, and SPS 61) used the commercial device Myo armband that has eight sensors with a corresponding sampling rate of 200 Hz, and the other six (SPS 5, SPS 25, SPS 27, SPS 59, SPS 62, and SPS 63) used homemade devices with a similar design to the Myo armband, their sampling rates are 1000 Hz, 960 Hz, 1000 Hz, 1000 Hz, 1200 Hz, and 1000 Hz, respectively.

Additionally, the EMG sampling rate of 16 HGR models (SPS 1, SPS 5, SPS 10, SPS 11, SPS 26, SPS 27, SPS 30, SPS 31, SPS 32, SPS 37, SPS 38, SPS 39, SPS 43, SPS 48, SPS 49, and SPS 55) is 1000 Hz because these SPS indicate that the sampling rate must be at least twice the highest frequency of the EMG, according to the Nyquist sampling theory, and approximately 95% of the signal power in the EMG is below 400–500 Hz [114,115,116]). Table 7 also shows the use of commercial devices, including the Myo armband from Thalmic Labs Inc., the MA300 from Motion Lab Systems Inc., the Bio Radio 150 from Cleveland Medical Devices Inc., the ME6000 from Mega Electronics Ltd., the Analog Front End (ADS1298) from Texas Instruments, the Telemyo 2400T G2 from Noraxon, and the EMG-USB2 from OT Bioelettronica. Furthermore, two models (SPS 43 and SPS 45) use high-density EMG sensors.

#### 3.2.2. Segmentation

**Figure 4:**
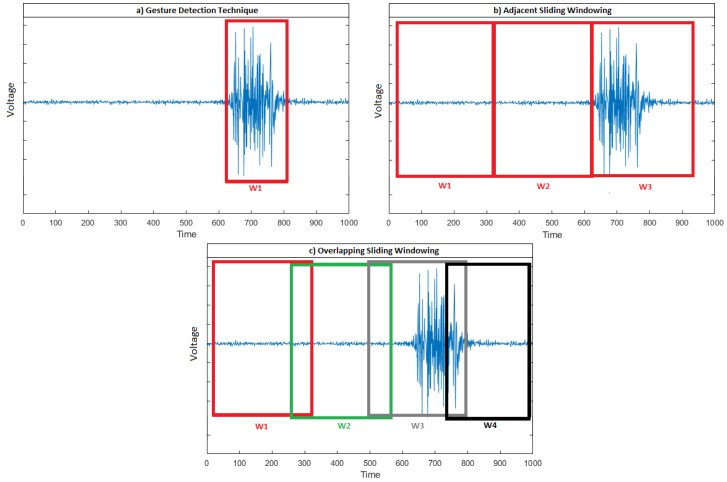
Segmentation of the EMG of a gesture using the three techniques: (**a**) gesture detection, (**b**) adjacent sliding windowing, and (**c**) overlapping sliding windowing.

EMGs are partitioned into multiple segments or windows using different techniques, such as gesture detection and sliding windowing (see Table 7). Gesture detection computes the beginning and the end of a hand gesture, and returns the EMG that only corresponds to muscle contraction. Therefore, the segment lengths are variable as they depend on the duration of the hand gestures. The sliding windowing techniques partition the EMG into fixed adjacent segments (i.e., adjacent sliding windowing) or fixed overlapping segments (i.e., overlapping sliding windowing) (see Figure 4). By increasing the window length, up to a certain point, the controller delay increases, and also the accuracy of the models increase as more data are collected for recognition [25,40].

#### 3.2.3. Preprocessing

HGR models use preprocessing techniques that transform the EMG into an input signal for Feature Extraction or for the ML algorithm if the structure of the HGR model does not have Feature Extraction (see Table 6). For example, a common preprocessing technique is the use of a Notch Filter at 50 or 60 Hz that eliminates the AC frequency of the powerlines (SPS 10). Other examples include Offset Compensation, Pre-smoothing, Filtering, Rectification, Amplification, and the use of the Teager–Kaiser-Energy Operator (see Table 7). Offset Compensation is a technique that eliminates noise through the compensation of the average value of the EMG:

(3) $$EMG_{raw}=\left(x_{1},x_{2},\ldots ,x_{n}\right)$$

(4) $$mean\left(EMG_{raw}\right)=\bar{x}=\frac{\sum_{i=1}^{n}\left(x_{i}\right)}{n}$$

(5) $$EMG_{offset}=\left(\left(x_{1}-\bar{x}\right),\left(x_{2}-\bar{x}\right),\ldots ,\left(x_{n}-\bar{x}\right)\right)$$

(6) $$mean(EMG_{offset})=0,$$

where, $x_{1},x_{2},\ldots ,x_{n}$ are the raw EMG values, $\bar{x}$ is the average value of the signal, and $\left(x_{1}-\bar{x}\right),\left(x_{2}-\bar{x}\right),\ldots ,\left(x_{n}-\bar{x}\right)$ are the EMG values after the use of offset compensation. Pre-smoothing is a technique that computes the mean of the last *m* values of the EMG and then sets the mean to the current value $x_{n}$ of the signal:

(7) $$EMG_{raw}=\left(x_{1},x_{2},\ldots ,x_{n}\right)$$

(8) $$x_{n}=\frac{\sum_{i=1+n-m}^{n}\left(x_{i}\right)}{m},$$

where, $x_{1},x_{2},\ldots ,x_{n}$ are the raw EMG values and $x_{n}$ is the current value that is based on the mean of the *m* previous values of the raw EMG. Filtering is a technique that removes some unwanted frequencies or an unwanted frequency band from the raw EMG. Rectification transforms the negative values into positive values (e.g., absolute value function). The Teager–Kaiser-Energy Operator increases the signal-to-noise ratio to improve the muscle activity onset detection of a gesture [117]. The most used preprocessing technique is filtering (see Table 7).

#### 3.2.4. Feature Extraction

Feature extraction techniques map the EMG into a feature set. These techniques extract features in different domains, such as time, frequency, time-frequency, space, and fractal. Table 8 shows the domains of the feature extraction techniques used by the models. Most of the real-time HGR models use time-domain features because the controller delay for their computation is less than the controller delay for the computation of features in other domains (see Table 9). The mean absolute value is the most used feature in the 65 studies analyzed.

#### 3.2.5. Classification

In this stage, classifiers generate class labels (i.e., the gestures recognized) from a feature set of the EMG. The classifiers used are support vector machines (SPS 7, SPS 10, SPS 14, SPS 15, SPS 18, SPS 23, SPS 25, SPS 27, SPS 28, SPS 30, SPS 38, SPS 39, SPS 49, SPS 52, SPS 53, SPS 55, and SPS 59), feedforward neural networks (SPS 2, SPS 16, SPS 17, SPS 22, SPS 24, SPS 26, SPS 29, SPS 32, SPS 35, SPS 36, SPS 44, SPS 42, SPS 46, SPS 47, SPS 56, SPS 60, and SPS 61), linear discriminant analysis (SPS 5, SPS 11, SPS 13, SPS 31, SPS 37, SPS 45, SPS 48, SPS 57, SPS 63, SPS 64, and SPS 65), convolutional neural networks (CNN) (SPS 4, SPS 20, SPS 43, and SPS 62), CNN with transfer learning (SPS 34), radial basis function networks (SPS 40), temporal convolutional networks (SPS 41), k-nearest neighbors and dynamic time warping (SPS 8, and SPS 9), collaborative-representation-based classification (SPS 19), k-nearest neighbors (SPS 1), k-nearest neighbors and decision trees (SPS 12), binary tree-support vector machine (SPS 21), vector auto-regressive hierarchical hidden Markov models (SPS 6), Gaussian mixture models and hidden Markov models (SPS 3), quadratic discriminant analysis (SPS 33), fuzzy logic (SPS 50), recurrent neural networks (SPS 51), generalized regression neural networks (SPS 54), and one vs one classifier (58). The most commonly used ML algorithms are support vector machines, feedforward neural networks, and linear discriminant analysis.

#### 3.2.6. Postprocessing

To improve the accuracy of the HGR models, the postprocessing techniques adapt the output of the ML algorithm to the final application. Only 15 out of 65 SPS used postprocessing techniques, such as majority voting (SPS 2, SPS 11, SPS 21, SPS 37 and SPS 43), elimination of consecutive repetitions (SPS 8, SPS 9, SPS 36, and SPS 51), threshold (SPS 35, and SPS 44), and velocity ramps (SPS 60, SPS 63, SPS 64, and SPS 65).

Many works perform an analysis of some of the stages shown in Section 3.2 to determine the best structure to improve the accuracy of the HGR models, for example, data acquisition [39,48,118,119], optimal window length [120], filtering [121,122], feature extraction [123], and classification [124,125] stages. However, the results are inconclusive because the structure of the HGR models depend on the environment in which the models are developed (i.e., the data sets used, the people who participated in the evaluation, the application of the models, etc.)

### 3.3. Results of the RQ2 (What Is the Controller Delay and Hardware Used by Real-Time HGR Models Using EMG and ML?)

#### 3.3.1. Controller Delay of the HGR Models

The controller delay is the sum of two values, which are the data collection time (DCT) (i.e., window length) and the data analysis time (DAT) [39,42]. In real-time processing, the DCT and DAT should be as short as possible, but the DCT also should allow the HGR model to collect enough EMG data to recognize a hand gesture. For instance, in prosthesis control, the optimal DCT using four EMG sensors with a sampling rate of 1 kHz should be between 150–250 ms [120].

An HGR model using EMG is considered to work in real-time when the response time (i.e., controller delay) is less than the optimal controller delay. There are several optimal controller delays reported in the scientific literature, namely 300 ms [39], 500 ms [41], and 100 ms for fast prosthetic prehensors and 125 ms for slower prosthetic prehensors [42].

In accordance with the Inclusion and Exclusion Criteria (see Section 2.3.1), all 65 HGR models indicate that they are real-time models. However, there are some SPS that did not report the controller delay (i.e., DCT and DAT) of their HGR models. Table 10 shows the DCT and DAT of the SPS.

#### 3.3.2. Hardware Used

The controller delay of the HGR models not only depends on their structure but also on the hardware used to process the models. For example, an HGR model may not work in real-time if the user perceives delays in the HGR response because the device has limited processing capabilities. The same HGR model may also be considered to work in real-time in another device with better processing capabilities. For this reason, when a model is described, it is fundamental to indicate the hardware characteristics of the devices used for running an HGR model. Table 10 shows the two types of hardware used, which are personal computers and embedded systems. Ten HGR models were processed in personal computers, such as laptops, desktops, etc., five HGR models were processed in embedded systems, and the remaining models did not indicate the hardware used.

### 3.4. Results of the RQ3 (What Is the Number and Type of Gestures Recognized by Real-Time HGR Models Using EMG and ML?)

#### 3.4.1. Number of Gestures Recognized

The number of gestures recognized is the number of classes of an HGR model. There are HGR models that have the same number of gestures, and each model has different gestures. For example, there are two HGR models that recognize four gestures, but the classes of the first model are thumb up, okay, wrist valgus, and wrist varus (SPS 14), and the classes of the second model are hand extension, hand grasp, wrist extension, and thumb flexion (SPS 22). Hence to compare these models, it is important to consider the difference in the gestures as well.

#### 3.4.2. Type of Gestures Recognized

The hand gestures, according to the type of movement, are classified as static and dynamic. A static gesture is made when the skeletal muscles are in constant contraction (i.e., there is no movement during the gesture), and in a dynamic gesture, the skeletal muscles are in contraction, but it is not constant, which indicates that there is movement during the gesture.

The EMG data generated by a gesture has two states: transient and steady. The EMG data in the transient state are generated during the transition from one gesture to another, and the EMG data in the steady state are generated when a gesture is maintained [38]. Moreover, the offline classification of hand gestures using EMG data in the steady state is more accurate than in the transient state as the variance of the EMG data in the transient state varies more (i.e., non-stationary process) than in the steady state over time [40]. However, in the training phase, the inclusion of EMG data in the transient state improves subject performance in a real-time virtual clothespin task [46,48].

**Figure 5:**
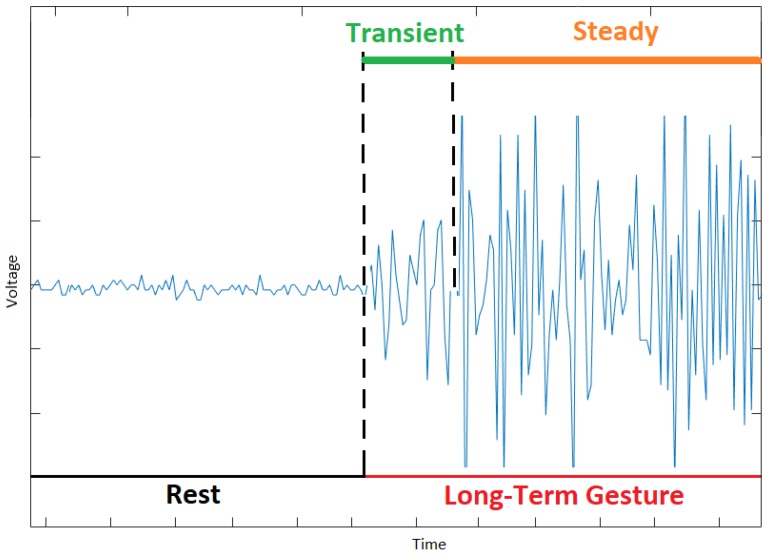
The EMG data of a long-term peace gesture (most of the EMG data are in the steady state).

**Figure 6:**
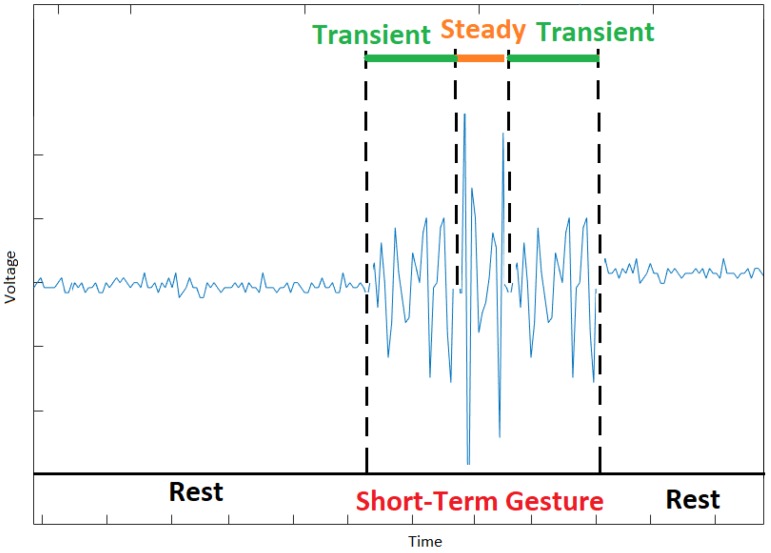
The EMG data of a short-term peace gesture (most of the EMG data are in the transient state).

Figure 5 presents the EMG data of a person who made a long-term gesture (i.e., gestures that lasted a long time) after a relaxed position or rest gesture. In this figure, the EMG data in the transient state are generated during the transition from the rest gesture to the peace sign, and the EMG data in the steady state are generated when the peace sign is maintained. The short-term gestures (i.e., gestures that lasted only a short time) generate more EMG data in the transient state than in the steady state as most of the time is spent in transitions from one gesture to another (see Figure 6).

The durations of the gestures used by the models are shown in Table 11. This table shows seven aspects about the gestures recognized by the HGR models reviewed in this SLR, such as the number of classes, the number of gestures per person in the training set (NGpPT), the number of people who participated in the training (NPT), the number of gestures per person in the evaluation set (NGpPE), the type of gestures recognized, the state of the EMG data used, and the duration of the gestures (DG). NGpPT, NPT, and DG show the EMG data used to train the individual (NGpPT×DG), and general (NGpPT×NPT×DG) models. We found that 63 out of 65 HGR models recognized static gestures, and only one HGR model recognized both dynamic and static gestures (SPS 25); moreover, no HGR model recognized only dynamic gestures. Additionally, six SPS used EMG data in the steady state, two SPS used EMG data in the transient state, three SPS used EMG data in the steady and transient states, and the remaining HGR models did not indicate the state of the EMG data. There were 31 out of the 65 HGR models that considered the rest gesture (i.e., the hand does not make any movement) as a class.

Finally, 5 out of the 65 HGR models (SPS 59, SPS 60, SPS 62, SPS 63, and SPS 64) recognized static gestures simultaneously to control multiple degrees of freedom of a prosthesis, which replicates simultaneous movements, such as wrist rotation and grasp to turn a doorknob. The remaining HGR models recognized gestures sequentially.

### 3.5. Results of the RQ4 (What Are the Metrics Used to Evaluate Real-Time HGR Models Using EMG and ML?)

According to the type of evaluation (see Section 1), we divide the SPS into two groups. HGR models evaluated using metrics for machine learning (56 models), and target achievement tests (nine models).

#### 3.5.1. HGR Models Evaluated Using Metrics for Machine Learning (from SPS 1 to SPS 56)

These 56 HGR models used 13 evaluation metrics (see Table 12), such as accuracy (9), recall (10), precision (11), accuracy per user (12), recall per user (13), precision per user (14), median of the accuracy per user (15), standard deviation of the accuracy per user (16), standard deviation of the accuracy per class (17), standard deviation of each user accuracy (18), standard deviation of the recalls of each class (19), classification error (20), and Kappa index (21). The accuracy is the metric most used, Table 12 shows the evaluation metrics used by these 56 models. The formulas of these evaluation metrics are:

(9) $$Accuracy=\frac{\sum_{i=1}^{u}\sum_{j,k=1}^{g}n_{i,j,k}}{\sum_{i=1}^{u}\sum_{j=1}^{g}\sum_{k=1}^{g}n_{i,j,k}}$$

(10) $$Recall_{class(k)}=\frac{\sum_{i=1}^{u}n_{i,k,k}}{\sum_{i=1}^{u}\sum_{j=1}^{g}n_{i,j,k}}$$

(11) $$Precision_{class(j)}=\frac{\sum_{i=1}^{u}n_{i,j,j}}{\sum_{i=1}^{u}\sum_{k=1}^{g}n_{i,j,k}}$$

(12) $$Accuracy_{user(i)}=\frac{\sum_{j,k=1}^{g}n_{i,j,k}}{\sum_{j=1}^{g}\sum_{k=1}^{g}n_{i,j,k}}$$

(13) $$Recall_{user(i)class(k)}=\frac{n_{i,k,k}}{\sum_{j=1}^{g}n_{i,j,k}}$$

(14) $$Precision_{user(i)class(j)}=\frac{n_{i,j,j}}{\sum_{k=1}^{g}n_{i,j,k}}$$

(15) $$Median(Accuracy_{user(1)},Accuracy_{user(2)},\ldots ,Accuracy_{user(u)})$$

(16) $$SD_{users}=\sqrt{\frac{\sum_{i=1}^{u}{\left(Accuracy_{user(i)}-Accuracy_{model}\right)}^{2}}{u-1}}$$

(17) $$SD_{classes}=\sqrt{\frac{\sum_{k=1}^{g}{\left(Recall_{class(k)}-Accuracy_{model}\right)}^{2}}{g-1}}$$

(18) $$SD_{user(i)}=\sqrt{\frac{\sum_{k=1}^{g}{\left(Recall_{user(i),class(k)}-Accuracy_{user(i)}\right)}^{2}}{g-1}}$$

(19) $$SD_{class(k)}=\sqrt{\frac{\sum_{i=1}^{u}{\left(Recall_{user(i),class(k)}-Recall_{class(k)}\right)}^{2}}{u-1}}$$

(20) AccuracyError=1−Accuracy

(21) $$KappaIndex=\frac{Accuracy-\left(\frac{1}{{\left(\sum_{i=1}^{u}\sum_{j=1}^{g}\sum_{k=1}^{g}n_{i,j,k}\right)}^{2}}\right)\times \sum_{i=1}^{u}\sum_{aux=1}^{g}\left(\sum_{k=1}^{g}n_{i,aux,k}\right)\times \left(\sum_{j=1}^{g}n_{i,j,aux}\right)}{1-\left(\frac{1}{{\left(\sum_{i=1}^{u}\sum_{j=1}^{g}\sum_{k=1}^{g}n_{i,j,k}\right)}^{2}}\right)\times \sum_{i=1}^{u}\sum_{aux=1}^{g}\left(\sum_{k=1}^{g}n_{i,aux,k}\right)\times \left(\sum_{j=1}^{g}n_{i,j,aux}\right)}$$

where $n_{i,j,k}$ is the number of gestures made by the user i, which were recognized by the model as *j* but they were *k*. $i\epsilon I=i_{1},i_{2},\ldots ,i_{u}$ is the set of test users, $j\epsilon J=j_{1},j_{2},\ldots ,j_{g}$ is the set of predicted classes, $k\epsilon K=k_{1},k_{2},\ldots ,k_{g}$ is the set of actual classes, *u* is the total number of test users, and *g* is the number of classes.

We identified five machine-learning metrics that evaluate the entire HGR model. The first one is accuracy, which is the fraction of gestures recognized correctly among all the test data. Second, the recall is the fraction of gestures recognized correctly for a class among the test data of this class. Third, the precision is the fraction of gestures recognized correctly of a class among the gestures recognized by the HGR model as this class. Fourth, the standard deviation of the accuracy per user is the amount of dispersion of the recognition accuracies per user. Finally, the standard deviation of the accuracy per class is the amount of dispersion of the recalls of a particular model.

These metrics can produce biased results for two reasons: an incorrect definition of a true positive, and an unbalanced test. In order to determine the recognition accuracy, a gesture is considered as a true positive (i.e., the gesture is recognized correctly) when the HGR model determines what gesture was performed and when this gesture was performed by a person. However, only SPS 51 is evaluated in this way. Eleven HGR models (SPS 2, SPS 5, SPS 6, SPS 7, SPS 8, SPS 9, SPS 19, SPS 20, SPS 34, SPS 35, and SPS 36) determine the classification accuracy because they only took into consideration what gesture was performed by a person as a true positive, and the remaining models do not show what they consider a true positive.

In addition, the test set is balanced when it has the same number of samples per class and the same number of samples per user (see Table 13). For example, if an HGR model is evaluated using a set that has more data for the user A, the accuracy of this model and the accuracy of the user A tend to be the same.

There are five SPS (SPS 2, SPS 5, SPS 8, SPS 9, and SPS 18) in which the evaluation was performed with data acquired without feedback (i.e., the correctness of classification was not provided in the evaluation), thus people cannot adjust their movements to the HGR model. Eight SPS were performed with data acquired with feedback from the HGR model (SPS 1, SPS 4, SPS 11, SPS 12, SPS 13, SPS 17, SPS 20, and SPS 29), and the remaining SPS do not indicate information about feedback.

Table 13 shows the recognition accuracies, the number of people who participated in the evaluation, type of data set (i.e., balanced or unbalanced), and the use of Cross-Validation by the 56 HGR models. The largest number of people is 80 (SPS 23). Three HGR models were evaluated using EMG data from amputees (SPS 6, SPS 21, and SPS 48). Moreover, 19 HGR models use cross-validation, that is, a technique used to minimize the probability of biased results in small data sets (see Table 13).

#### 3.5.2. HGR Models Evaluated Using Target Achievement Tests (from SPS 57 to SPS 65)

These nine HGR models used three target achievement tests, including the motion test (SPS 60), target achievement control test (TAC) (SPS 60, SPS 63, and SPS 65), and Fitts’ law test (FLT) (SPS 59, SPS 61, SPS 62, SPS 64, and SPS 65). These three tests used ten metrics, such as throughput (SPS 57, SPS 58, SPS 59, SPS 61, SPS 62, SPS 64, and SPS 65), path efficiency (SPS 57, SPS 58, SPS 59, SPS 60, SPS 61, SPS 62, SPS 64, and SPS 65), overshoot (SPS 57, SPS 58, SPS 59, SPS 61, SPS 62, SPS 64, and SPS 65), average speed (SPS 57), completion rate (SPS 57, SPS 58, SPS 60, SPS 61, SPS 63, SPS 64, and SPS 65), stopping distance (SPS 58), completion time (SPS 60, SPS 63, and SPS 65), real-time accuracy (SPS 60), length error (SPS 63), and reaction time (SPS 64) (see Table 14).

A motion test was proposed by patients with targeted muscle reinnervation to evaluate the myoelectric capacity [128]. These patients should maintain a gesture until the HGR model has made a predetermined number of correct predictions. In TAC, the patients control a virtual prosthesis to obtain a target for a dwell time, which is generally 1 s [50]. These patients have a trial time to get the target, which is generally 15 s. FLT is a similar test to TAC, but the users control a circular cursor with two or three degrees of freedom. FLT states that there is a trade-off between speed and accuracy [51,108], which is defined by:

(22) MT=a+b∗ID

where MT is the movement time, *a* and *b* are empirical constants, and ID is the index of difficulty (ID) of a target (see Equation (23)), which is calculated using the distance (D) from an initial point to a target, and the width (W) of the target. Throughput is a metric proposed by Fitts, which is the ratio between the ID and MT (see Equation (24)), to summarizes the performance of a control system. The results of FLT are reliable when this test combines a variety of IDs [129].

(23) $$ID=\log_{2}\left(\frac{D}{W}+1\right)$$

(24) $$TP=\frac{ID}{MT}$$

The people who participated in these tests received feedback (i.e., the correctness of classification was provided in the evaluation). Four out of these nine HGR models were evaluated with four amputees (SPS 63), two amputees (SPS 59, and SPS 64), and one amputee (SPS 65).

In order to achieve concluding results, it is necessary to consider the sample size, which is the number of people who participated in the evaluation $\left(n_{1}\right)$ (see Table 11) times the number of gestures per person $\left(n_{2}\right)$ (see Table 13), to allow us to obtain statistically significant results. Using the typical values of a statistical hypothesis test (confidence level of 95%, margin of error of 5%, and population portion of 50%), we estimated $n_{1}$ according to the Normal Distribution using the Central Limit Theorem (25), and $n_{2}$ according to the Hoeffding’s inequality (26), which is widely used in machine learning theory.

(25) $$n_{1}\geq \frac{z^{2}*p*\left(1-p\right)}{\epsilon^{2}}=\frac{1.96^{2}*0.5*\left(1-0.5\right)}{0.05^{2}}\approx 385$$

(26) $$n_{2}\geq -\frac{ln\frac{\left(1-\alpha \right)}{2}}{2\epsilon^{2}}=-\frac{ln\frac{\left(1-0.95\right)}{2}}{2*0.05^{2}}\approx 738,$$

where, *z* is the critical value of the normal distribution for a confidence level of 95%, ϵ is the margin of error, *p* is the population portion, and α is the confidence level. Therefore, the sample size $\left(n_{1}*n_{2}\right)$ gestures of the test set must be in the order of hundreds of thousands. None of the works present so far considered these values to achieve a significant result. In the scientific literature, many EMG data sets are available [130], but, according to the best of our knowledge, the data set with the higher $n_{1}$ is 30 [131], and with the higher $n_{2}$ is 40 [84,132].

## 4. Conclusions

This SLR analyzes works that propose HGR models using surface EMG and ML. Following the Kitchenham methodology, we introduced four RQs based on the main goal of this SLR, which was to analyze the state-of-the-art of these models. To answer these four RQs, we presented, analyzed, and discussed the data extracted from 65 selected primary studies. Below are our findings in regard to the four RQs.

**Structure:** The structure of the models studied varies from one work to the other. However, we were able to examine the structure of these models using a structure composed of six stages: data acquisition, segmentation, preprocessing, feature extraction, classification, and postprocessing. Under this standard structure, we studied the types of HGR models, the number of EMG sensors, the sampling rate, sensors, segmentation and preprocessing techniques, extracted features, the domain of the extracted features, and the ML algorithm. The most used structure is: eight EMG sensors, a sampling rate between 200 Hz and 1000 Hz, overlapping sliding windowing, filtering (segmentation), mean absolute value (feature extraction), support vector machines, and feedforward neural networks (classification).

**Controller delay and hardware:** The controller delay of gesture recognition models is the sum of two values: data collection time (DCT) and data analysis time (DAT). A recognition model works in real-time when this sum is less than an optimal controller delay. However, the works analyzed report several optimal controller delays for different applications, suggesting that the optimal controller delay is relative to the user perception and the application of a recognition model.

**Number and types of gestures recognized:** The 65 works analyzed propose models that recognize different number and types of gestures: 31 works took into consideration the rest gesture as a class to be recognized; only one model recognized both static and dynamic gestures; and the remaining models recognized static gestures only. No model recognized dynamic gestures only as most of the EMG data generated by dynamic gestures are in the transient state. Recognizing gestures using EMG data in the transient state is more complex than in the steady state because the latter behaves as a non-stationary process. The classification of the hand gestures using EMG data in the steady state is more accurate than in the transient state, and only nine works recognized short-term gestures (i.e., using EMG data in the transient state).

**Metrics and results:** We divided the SPS according to the types of evaluation, which are machine-learning metrics and target achievement tests. 56 SPS evaluated their models using machine learning metrics. We found 13 machine-learning metrics and three target achievement tests. The training and testing protocols vary among the works making the comparison of their performance very difficult. Moreover, taking into consideration that many works do not describe these protocols and the whole structure of the model, one key point is the significance and reproducibility of the results. Using the normal distribution for the number of people, and the Hoeffding’s inequality for the number of gestures per person, we estimated that the sample size of the test set must be in the order of the hundreds of thousands to obtain a result with a confidence level of 95% and a precision of 5%. None of the works analyzed utilize a test set of this magnitude, and therefore the confidence and reproducibility of their results are questionable. Based on the definition a true positive, only one out of the HGR models, which used machine-learning metrics, was evaluated using the recognition accuracy; the remaining models were evaluated using classification accuracy as they only took into consideration what gesture was performed by a person as a true positive.

## 5. Future Work

Based on this SLR, we identify the possible future works in this field:

- Research the optimal permitted delay to determine a general criterion of real-time processing in HGR models using EMG and ML.
- Develop models using EMG and ML to recognize gestures of long and short duration. Therefore, these models must be able to recognize gestures using EMG data in the transient and steady states.
- Develop evaluation methods for the HGR models using EMG and ML that state the test sets, metrics, and protocol of evaluation.
- Develop general HGR models using EMG and ML that can be used by people who do not participate in the training of these models.
- Develop recognition models that not only recognize one gesture but a sequence of movements.

## Figures and Tables

**Table 1:** Search strings used to find primary studies.

ID	Search String
SS1	“Electromyography” AND “Hand Gesture Recognition” AND “Real Time”
SS2	“Electromyography” AND “Hand Gesture Recognition” AND “Real-Time”
SS3	“Electromyography” AND “Hand Gesture Recognition” AND “Online”
SS4	“Electromyography” AND “Hand Gesture Recognition” AND “On line”
SS5	“Electromyography” AND “Hand Gesture Recognition” AND “On-line”
SS6	“Electromyography” AND “Hand Gesture Recognition” AND “box and blocks”
SS7	“Electromyography” AND “Hand Gesture Recognition” AND “target achievement control”
SS8	“Electromyography” AND “Hand Gesture Recognition” AND “Fitts’ law”
SS9	“EMG” AND “Hand Gesture Recognition” AND “Real Time”
SS10	“EMG” AND “Hand Gesture Recognition” AND “Real-Time”
SS11	“EMG” AND “Hand Gesture Recognition” AND “Online”
SS12	“EMG” AND “Hand Gesture Recognition” AND “On line”
SS13	“EMG” AND “Hand Gesture Recognition” AND “On-line”
SS14	“EMG” AND “Hand Gesture Recognition” AND “box and blocks”
SS15	“EMG” AND “Hand Gesture Recognition” AND “target achievement control”
SS16	“EMG” AND “Hand Gesture Recognition” AND “Fitts’ law”

**Table 2:** Number of primary studies for each literature repository and search string.

Literature Repositories	Search Strings (SS)															
	SS1	SS2	SS3	SS4	SS5	SS6	SS7	SS8	SS9	SS10	SS11	SS12	SS13	SS14	SS15	SS16
IEEE Xplore	66	66	7	13	3	50	50	3	6	1	4	5	46	53	12	12
ACM Digital Library	34	34	5	13	13	33	33	1	13	13	25	30	68	81	2	2
Science Direct	34	34	25	25	25	41	41	30	30	30	1	1	3	3	3	3
Springer	52	52	29	7	7	75	75	29	9	9	3	3	2	2	2	2

**Table 3:** The identifier, title, and reference of the 65 selected primary studies (SPS) used in this SLR.

ID SPS	Title	Type of Publication
SPS 1	A Bionic Hand Controlled by Hand Gesture Recognition Based on Surface EMG Signals: A Preliminary Study [1]	Journal
SPS 2	Real-Time Hand Gesture Recognition Based on Electromyographic Signals and Artificial Neural Networks [55]	Conference
SPS 3	sEMG-Based Continuous Hand Gesture Recognition Using GMM-HMM and Threshold Model [56]	Conference
SPS 4	Hand Gestures Recognition Using Machine Learning for Control of Multiple Quadrotors [57]	Symposium
SPS 5	Real-Time Myocontrol of a Human–Computer Interface by Paretic Muscles After Stroke [58]	Journal
SPS 6	Decoding of Individual Finger Movements From Surface EMG Signals Using Vector Autoregressive Hierarchical Hidden Markov Models (VARHHMM) [59]	Conference
SPS 7	User-Independent Real-Time Hand Gesture Recognition Based on Surface Electromyography [60]	Conference
SPS 8	Hand Gesture Recognition Using Machine Learning and the Myo Armband [61]	Conference
SPS 9	Real-Time Hand Gesture Recognition Using the Myo Armband and Muscle Activity Detection [62]	Conference
SPS 10	A Sub-10 mW Real-Time Implementation for EMG Hand Gesture Recognition Based on a Multi-Core Biomedical SoC [63]	Workshop
SPS 11	Design and Myoelectric Control of an Anthropomorphic Prosthetic Hand [3]	Journal
SPS 12	Wearable Armband for Real Time Hand Gesture Recognition [64]	Conference
SPS 13	Simple Space-Domain Features for Low-Resolution sEMG Patternn Recognition [65]	Conference
SPS 14	A Wireless Surface EMG Acquisition and Gesture Recognition System [66]	Congress
SPS 15	Single Channel Surface EMG Control of Advanced Prosthetic Hands: A Simple, Low Cost and Efficient Approach [2]	Journal
SPS 16	The Virtual Trackpad: an Electromyography-Based, Wireless, Real-Time, Low-Power, Embedded Hand Gesture Recognition System Using an Event-Driven Artificial Neural Network [67]	Journal
SPS 17	Muscle-Gesture Robot Hand Control Based on sEMG Signals With Wavelet Transform Features and Neural Network classifier [68]	Conference
SPS 18	Evaluating Sign Language Recognition Using the Myo Armband [69]	Symposium
SPS 19	Spectral Collaborative Representation Based Classification for Hand Gestures Recognition on Electromyography Signals [70]	Conference
SPS 20	A Convolutional Neural Network for Robotic Arm Guidance Using sEMG Based Frequency-Features [71]	Conference
SPS 21	EMG Pattern Recognition Using Decomposition Techniques for Constructing Multiclass Classifier [72]	Conference
SPS 22	SEMG Based Human Computer Interface for Physically Challenged Patients [73]	Conference
SPS 23	EMG Feature Set Selection Through Linear Relationship for Grasp Recognition [74]	Journal
SPS 24	A Portable Artificial Robotic Hand Controlled by EMG Signal Using ANN Classifier [75]	Conference
SPS 25	Real-Time American Sign Language Recognition System by Using Surface EMG Signal [5]	Conference
SPS 26	Hand Motion Recognition From Single Channel Surface EMG Using Wavelet & Artificial Neural Network [76]	Conference
SPS 27	A Versatile Embedded Platform for EMG Acquisition and Gesture Recognition [77]	Journal
SPS 28	Hybrid EMG classifier Based on HMM and SVM for Hand Gesture Recognition in Prosthetics [78]	Conference
SPS 29	Human–Computer Interaction System Design Based on Surface EMG Signals [79]	Conference
SPS 30	Towards EMG Control Interface for Smart Garments [80]	Symposium
SPS 31	Identification of Low Level sEMG Signals for Individual Finger Prosthesis [81]	Conference
SPS 32	Pattern Recognition of Eight Hand Motions Using Feature Extraction of Forearm EMG Signal [82]	Journal
SPS 33	Pattern Recognition of Number Gestures Based on a Wireless Surface EMG System [83]	Journal
SPS 34	Deep Learning for Electromyographic Hand Gesture Signal Classification Using Transfer Learning [84]	Journal
SPS 35	Real-Time Hand Gesture Recognition Model Using Deep Learning Techniques and EMG Signals [85]	Conference
SPS 36	Real-Time Hand Gesture Recognition Based on Artificial Feed-Forward Neural Networks and EMG [86]	Conference
SPS 37	Pattern Recognition-Based Real Time Myoelectric System for Robotic Hand Control [87]	Conference
SPS 38	Hand Gesture Recognition and Classification Technique in Real-Time [88]	Conference
SPS 39	Forearm Muscle Synergy Reducing Dimension of the Feature Matrix in Hand Gesture Recognition [89]	Conference
SPS 40	EMG Wrist-Hand Motion Recognition System for Real-Time Embedded Platform [90]	Conference
SPS 41	Robust Real-Time Embedded EMG Recognition Framework Using Temporal Convolutional Networks on a Multicore IoT Processor [91]	Journal
SPS 42	A Multi-Gestures Recognition System Based on Less sEMG Sensors [92]	Conference
SPS 43	A Fully Embedded Adaptive Real-Time Hand Gesture Classifier Leveraging HD-sEMG & Deep Learning [93]	Journal
SPS 44	Real-time Pattern Recognition for Hand Gesture Based on ANN and Surface EMG [94]	Conference
SPS 45	Adjacent Features for High-Density EMG Pattern Recognition [95]	Conference
SPS 46	Automatic EMG-based Hand Gesture Recognition System Using Time-Domain Descriptors and Fully-Connected Neural Networks [96]	Conference
SPS 47	Artificial Neural Network to Detect Human Hand Gestures for a Robotic Arm Control [97]	Conference
SPS 48	Electromyography-Based Hand Gesture Recognition System for Upper Limb Amputees [98]	Journal
SPS 49	Robust Hand Gesture Recognition With a Double Channel Surface EMG Wearable Armband and SVM classifier [99]	Journal
SPS 50	Fuzzy Classification of Hand’s Motion [100]	Conference
SPS 51	EMG-Based Online Classification of Gestures With Recurrent Neural Networks [101]	Journal
SPS 52	Teleoperated Robotic Arm Movement Using Electromyography Signal With Wearable Myo Armband [102]	Journal
SPS 53	Identification of Gesture Based on Combination of Raw sEMG and sEMG Envelope Using Supervised Learning and Univariate Feature Selection [103]	Journal
SPS 54	Surface EMG Hand Gesture Recognition System Based on PCA and GRNN [104]	Journal
SPS 55	Dexterous Hand Gestures Recognition Based on Low-Density sEMG Signals for Upper-Limb Forearm amputees [105]	Journal
SPS 56	Real-Time Surface EMG Pattern Recognition for Hand Gestures Based on an Artificial Neural Network [106]	Journal
SPS 57	On the Usability of Intramuscular EMG for Prosthetic Control: A Fitts’ Law Approach [107]	Journal
SPS 58	Validation of a Selective Ensemble-Based Classification Scheme for Myoelectric Control Using a Three-Dimensional Fitts’ Law Test [108]	Journal
SPS 59	Support Vector Regression for Improved Real-Time, Simultaneous Myoelectric Control [109]	Journal
SPS 60	Real-Time and Simultaneous Control of Artificial Limbs Based on Pattern Recognition Algorithms [110]	Journal
SPS 61	On the Robustness of Real-Time Myoelectric Control Investigations: A Multiday Fitts’ Law approach [111]	Journal
SPS 62	Regression Convolutional Neural Network for Improved Simultaneous EMG Control [112]	Journal
SPS 63	A Comparison of the Real-Time Controllability of Pattern Recognition to Conventional Myoelectric Control for Discrete and Simultaneous Movements [31]	Journal
SPS 64	A Real-Time Comparison Between Direct Control, Sequential Pattern Recognition Control and Simultaneous Pattern Recognition Control Using a Fitts’ Law Style Assessment Procedure [113]	Journal
SPS 65	Evaluation of Computer-Based Target Achievement Tests for Myoelectric Control [46]	Journal

**Table 4:** Inclusion and exclusion criteria used in this systematic literature review (SLR).

**Inclusion**	Primary studies about the development of the Hand Gesture Recognition (HGR) model.
**Criteria**	Primary studies that use electromyography (EMG) as input of the HGR model.
	The full text of the primary study was not available.
**Exclusion**	Primary studies that do not use machine learning (ML) in the HGR model.
**Criteria**	Primary studies that do not indicate that their models are in real time.
	Primary studies that are in another language than English.
	Primary studies that are not peer-reviewed.

**Table 5:** The data extracted from the 65 SPS and their targets.

Extracted Data	Target
Publication year	Study overview
Primary study type	Study overview
Structure of the HGR model	RQ1
Controller delay of the HGR model	RQ2
Hardware used	RQ2
Number of gestures recognized	RQ3
Types of gestures recognized	RQ3
Metrics and results used to evaluate the HGR models	RQ4

**Table 6:** Standard structure used by the 65 HGR models.

ID SPS	Stages of the Standard Structure					
	DA	SEGM	PREP	FE	CL	POSTP
SPS 1	yes	yes	no	yes	yes	no
SPS 2	yes	yes	yes	yes	yes	yes
SPS 3	yes	yes	no	yes	yes	no
SPS 4	yes	yes	no	no	yes	no
SPS 5	yes	yes	no	yes	yes	no
SPS 6	yes	yes	no	yes	yes	no
SPS 7	yes	yes	no	yes	yes	no
SPS 8	yes	yes	yes	no	yes	yes
SPS 9	yes	yes	yes	no	yes	yes
SPS 10	yes	yes	yes	yes	yes	no
SPS 11	yes	yes	yes	yes	yes	yes
SPS 12	yes	no	yes	yes	yes	no
SPS 13	yes	yes	no	yes	yes	no
SPS 14	yes	no	yes	yes	yes	no
SPS 15	yes	yes	yes	yes	yes	no
SPS 16	yes	yes	yes	yes	yes	no
SPS 17	yes	no	yes	yes	yes	no
SPS 18	yes	no	no	yes	yes	no
SPS 19	yes	yes	no	yes	yes	no
SPS 20	yes	yes	no	yes	yes	no
SPS 21	yes	yes	yes	yes	yes	yes
SPS 22	yes	no	yes	yes	yes	no
SPS 23	yes	no	yes	yes	yes	no
SPS 24	yes	no	no	yes	yes	no
SPS 25	yes	yes	yes	yes	yes	no
SPS 26	yes	yes	no	yes	yes	no
SPS 27	yes	yes	yes	no	yes	no
SPS 28	yes	no	yes	no	yes	no
SPS 29	yes	no	yes	yes	yes	no
SPS 30	yes	yes	yes	no	yes	no
SPS 31	yes	yes	yes	yes	yes	no
SPS 32	yes	yes	yes	yes	yes	no
SPS 33	yes	yes	yes	yes	yes	no
SPS 34	yes	yes	no	yes	yes	no
SPS 35	yes	yes	yes	no	yes	yes
SPS 36	yes	no	yes	yes	yes	no
SPS 37	yes	yes	yes	yes	yes	no
SPS 38	yes	no	yes	yes	yes	no
SPS 39	yes	yes	yes	yes	yes	no
SPS 40	yes	yes	no	yes	yes	no
SPS 41	yes	yes	yes	no	yes	yes
SPS 42	yes	yes	yes	yes	yes	yes
SPS 43	yes	yes	yes	yes	yes	no
SPS 44	yes	yes	no	yes	yes	no
SPS 45	yes	yes	no	no	yes	no
SPS 46	yes	yes	no	yes	yes	no
SPS 47	yes	no	yes	no	yes	yes
SPS 48	yes	yes	yes	yes	yes	yes
SPS 49	yes	yes	no	yes	yes	no
SPS 50	yes	yes	yes	yes	yes	no
SPS 51	yes	yes	yes	yes	yes	no
SPS 52	yes	no	no	yes	yes	yes
SPS 53	yes	yes	no	yes	yes	no
SPS 54	yes	yes	yes	yes	yes	no
SPS 55	yes	no	no	yes	yes	no
SPS 56	yes	yes	yes	yes	yes	no
SPS 57	yes	yes	yes	yes	yes	no
SPS 58	yes	yes	yes	no	yes	no
SPS 59	yes	yes	yes	yes	yes	no
SPS 60	yes	yes	no	yes	yes	yes
SPS 61	yes	yes	no	yes	yes	no
SPS 62	yes	yes	yes	no	yes	no
SPS 63	yes	yes	yes	yes	yes	yes
SPS 64	yes	yes	yes	yes	yes	yes
SPS 65	yes	yes	yes	yes	yes	yes

**yes**: The model used this stage; **no**: The model did not use this stage; **DA**: Data Acquisition Stage; **SEGM**: Segmentation Stage; **PREP**: Preprocessing Stage; **FE**: Feature Extraction Stage; **CL**: Classification stage; **POSTP**: Postprocessing Stage.

**Table 7:** The number of sensors, sampling rate, acquisition devices, segmentation techniques, and preprocessing techniques used in the 65 HGR models.

ID SPS	Number of Sensors	Sampling Rate (Hz)	Acquisition Device Used	Segmentation Technique Used	Preprocessing Techinique Used
SPS 1	2	1000	MA300	ASW	NI
SPS 2	8	200	Myo armband	OSW	FL and RE
SPS 3	8	200	Myo armband	OSW and GD	NI
SPS 4	8	200	Myo armband	ASW	NI
SPS 5	8	1000	Homemade device	OSW	NI
SPS 6	16	1600	Homemade device	OSW	NI
SPS 7	8	200	Myo armband	OSW and GD	NI
SPS 8	8	200	Myo armband	OSW	FL andRE
SPS 9	8	200	Myo armband	OSW andGD	FL and RE
SPS 10	3	1000	Homemade device	ASW	FL and OC
SPS 11	2	1000	Homemade device	ASW	PreS
SPS 12	3	NI	Homemade device	NI	FLandRE
SPS 13	8	200	Myo armband	OSW	NI
SPS 14	3	NI	Homemade device	NI	FL
SPS 15	1	NI	Homemade device	ASW and GD	RE
SPS 16	4	1600	Homemade device	ASW and GD	RE
SPS 17	8	200	Myo armband	NI	FL
SPS 18	8	200	Myo armband	NI	NI
SPS 19	8	200	Myo armband	OSW	NI
SPS 20	8	200	Myo armband	OSW	NI
SPS 21	16	1600	Homemade device	OSW	FL
SPS 22	1	125	Homemade device	NI	FL
SPS 23	2	NI	Homemade device	NI	FL
SPS 24	3	NI	Homemade device	NI	NI
SPS 25	8	960	Bio Radio 150	ASW	FL
SPS 26	1	1000	Homemade device	ASW	NI
SPS 27	8	1000	Homemade device	GD	FL
SPS 28	4	500	Homemade device	NI	FL
SPS 29	4	NI	Homemade device	NI	FL
SPS 30	4	1000	Homemade device	OSW and GD	FL, OC and RE
SPS 31	4	1000	Homemade device	OSW	OC
SPS 32	4	1000	Homemade device	ASW	FL
SPS 33	4	500	Homemade device	ASW and GD	FL
SPS 34	8	200	Myo armband	OSW	NI
SPS 35	8	200	Myo armband	OSW and GD	RE
SPS 36	8	200	Myo armband	OSW	FL and RE
SPS 37	2	1000	Homemade device	OSW and GD	FL and AMPL
SPS 38	1	1000	Homemade device	OSW	FL
SPS 39	6	1000	ME6000	NI	FL
SPS 40	8	200	Myo armband	OSW	NI
SPS 41	8	4000	Analog Front End (ADS1298)	OSW	NI
SPS 42	2	NI	Telemyo 2400T G2	ASW	NI
SPS 43	32	1000	Homemade device	NI	FL, RE and TKEO
SPS 44	8	200	Myo armband	ASW	FL and RE
SPS 45	128	2048	EMG-USB2	OSW	FL
SPS 46	8	200	Myo armband	ASW	NI
SPS 47	8	200	Myo armband	OSW	FL and NORM
SPS 48	8	1000	Analog Front End (ADS1298)	OSW	FL
SPS 49	2	1000	Homemade device	NI	FL and NORM
SPS 50	4	NI	Homemade device	GD	FL and AMPL
SPS 51	16	200	Myo armband	NI	NI
SPS 52	8	200	Myo armband	OSW	NI
SPS 53	2	2000	Homemade device	ASW and GD	FL and RE
SPS 54	16	NI	Homemade device	NI	NI
SPS 55	4	1000	Homemade device	OSW	NI
SPS 56	8	200	Myo armband	OSW	FL and RE
SPS 57	4	1000	Homemade device	OSW	FL
SPS 58	6	1000	Homemade device	OSW	FL
SPS 59	8	1000	Homemade device	OSW	FL
SPS 60	4	2000	Homemade device	OSW	NI
SPS 61	8	200	Myo armband	OSW	NI
SPS 62	8	1200	Homemade device	OSW	FL
SPS 63	8-12	1000	Homemade device	OSW	FL
SPS 64	6	1000	Homemade device	OSW	FL
SPS 65	4	200	Homemade device	OSW	FL

**NI**: Not indicated; **OSW**: Overlapping Sliding Windowing; **ASW**: Adjacent Sliding Windowing; **GD**: Gesture Detection; **FL**: Filtering; **RE**: Rectification; **OC**: Offset Compensation; **PreS**: Pre-smoothing; **AMPL**: Amplification; **TKEO**: Teager-Kaiser-Energy Operator; **NORM**: Normalization.

**Table 8:** Features according to the domain.

**Time-Domain Features**	Mean absolute value (MAV), root mean square (RMS), waveform length (WL), zero crossings (ZC), fourth-order autoregressive coefficients (AR-Coeff), standard deviation (SD), variance (VAR), slope sign changes (SSC), mean, median, integrated EMG (iEMG), sample entropy (SampEn), mean absolute value ratio (MAVR), modified mean absolute value (MMAV), simple square integral (SSI), Log detector (LOG), average amplitude change (AAC), maximum fractal length (MFL), minimum (MIN), maximum (MAX), Hjorth parameters (HJP), peak value (PK), energy ratio (ER), histogram (HISTG), willison amplitude (WAMP), kurtosis (KURT), skewness (SKEW), non-negative matrix factorization (NMF), natural logarithm of the variance (ln-VAR), root sum square (RSS), logarithm of the root mean square (log-RMS), logarithm of the integrated EMG (log-iEMG), logarithm of the variance (log-VAR), logarithmic band power (LBP), first derivation (DIFF), detrended fluctuation analysis (DFA), modified mean absolute values (MAV1-MAV2), V-order, difference absolute standard deviation value (DASDV), max-min, autoregressive model intercept (Inpt), cardinality (CARD)
**Frequency-Domain Features**	Amplitude spectrum (AmpSpec), mean frequency (MNF), median frequency (MDF), modified median frequency (MMDF), modified mean frequency (MMNF), mean power (MNP), cepstral coefficients (Cep-Coeff), circulant matrix structure for eigenvalue decomposition (CMSED), fast Fourier transform (FFT), median amplitude spectrum (MAS), peak frequency (PKF), total power (TTP), power spectrum ratio (PSR)
**Time-Frequency-Domain Features**	Discrete wavelet transform (DWT), continuous wavelet transform (CWT), mean of the absolute wavelet coefficients (MOAC), average power of the wavelet coefficients (APOC), standard deviation of the wavelet coefficients (STDOC), MOAC-ratio
**Space-Domain Features**	Scaled mean absolute value (SMAV), mean absolute difference of the normalized values (MADN)
**Fractal-Domain Features**	De-trended fluctuation analysis (DFA), Higuchi fractal dimension (HFD)

**Table 9:** Features used in the 65 HGR models.

ID SPS	Features used
SPS 1	MAV
SPS 2	MAV, RMS, WL, SSC, and HJP
SPS 3	RMS
SPS 4	NI
SPS 5	MAV, WL, ZC, and SSC
SPS 6	MAV
SPS 7	MAV, RMS, ZC, VAR, ER, HISTG, WAMP, AmpSpec, MMDF, and MMNF
SPS 8	NI
SPS 9	NI
SPS 10	RMS
SPS 11	MAV, AR-Coeff, VAR, and SampEn
SPS 12	WL, VAR, iEMG, and PK
SPS 13	SMAV, and MADN
SPS 14	AR-Coeff, and Mean
SPS 15	Mean
SPS 16	MAV
SPS 17	MAV, SD, and DWT
SPS 18	MAV
SPS 19	CMSED
SPS 20	FFT
SPS 21	MAV, WL, ZC, and SSC
SPS 22	RMS, SD, and SampEn
SPS 23	DWT
SPS 24	iEMG
SPS 25	MAV, RMS, SD, MMAV, SSI, LOG, AAC, MFL, MIN, and MAX
SPS 26	DWT
SPS 27	NI
SPS 28	NI
SPS 29	AR-Coeff
SPS 30	NI
SPS 31	MAV, RMS, MNP, and DFA
SPS 32	DWT
SPS 33	MAV, WL, ZC, and MAVR
SPS 34	CWT
SPS 35	NI
SPS 36	NI
SPS 37	RMS, WL, WAMP, SampEn, and Cep-Coeff
SPS 38	Mean, VAR, KURT, and SKEW
SPS 39	NMF
SPS 40	iEMG, ln-VAR, and RSS
SPS 41	NI
SPS 42	log-RMS, log-iEMG, log-VAR
SPS 43	NI
SPS 44	MAV, RMS, SSC, WL, and HJP
SPS 45	SMAV, and MADN
SPS 46	MAV, ZC, SSC, SKEW, RMS, HJP, and iEMG
SPS 47	RMS, and Median
SPS 48	RMS, WL, ZC, and SSC
SPS 49	Mean
SPS 50	RMS, LBP, and DIFF
SPS 51	SD
SPS 52	MAV, WL, RMS, AR-Coeff, ZC, and SSC
SPS 53	MAV, MAV1-MAV2, VAR, RMS, SSI, V-order, iEMG, DASDV, AAC, ZC, LOG, SSC, WL, WAMP, MFL, MAX, MIN, max-min, SKEW, KURT, TTP, MNF, MDF, MNP, PKF, MOAC, APOC, STDOC, MOAC-ratio, Inpt, AR-Coeff
SPS 54	RMS, WL, MAS and SampEn
SPS 55	MAV, MAV1, MAV2, VAR, RMS, WL, ZC, SSC, AR-Coeff, MNF, MDF, PKF, MNP, TTP, PSR, DFA, and HFD
SPS 56	MAV, SSC, WL, RMS, and HJP
SPS 57	MAV, WL, ZC, and SSC
SPS 58	NI
SPS 59	MAV, WL, ZC, and SSC
SPS 60	MAV, WL, ZC, and SSC
SPS 61	MAV, WL, ZC, SSC, WAMP, and CARD
SPS 62	NI
SPS 63	MAV, WL, ZC, and SSC
SPS 64	MAV, WL, ZC, and SSC
SPS 65	MAV, WL, ZC, and SSC

**Table 10:** Time of data collection and data analysis, and hardware used in the 65 HGR models.

ID SPS	DCT(ms)	DAT(ms)	Hardware Used
SPS 1	250	NI	NI
SPS 2	1000	29.38	Personal computer
SPS 3	100	37.9	Personal computer
SPS 4	250	NI	NI
SPS 5	250	NI	NI
SPS 6	250	NI	NI
SPS 7	300	500	NI
SPS 8	1000	250	Personal Computer
SPS 9	1000	193.1	Personal Computer
SPS 10	72	41	Embedded System
SPS 11	250	70	NI
SPS 12	NI	NI	NI
SPS 13	200	NI	NI
SPS 14	NI	NI	NI
SPS 15	NI	10	NI
SPS 16	250	0.2	Embedded System
SPS 17	NI	NI	Personal Computer
SPS 18	NI	NI	NI
SPS 19	500	NI	NI
SPS 20	285	15	Personal Computer
SPS 21	250	7.57	Personal Computer
SPS 22	NI	NI	NI
SPS 23	250	NI	NI
SPS 24	NI	NI	Embedded System
SPS 25	2000	NI	NI
SPS 26	NI	NI	NI
SPS 27	NI	NI	Embedded System
SPS 28	NI	NI	Embedded System
SPS 29	NI	NI	NI
SPS 30	NI	2.5	Personal Computer
SPS 31	250	NI	Personal Computer
SPS 32	256	NI	NI
SPS 33	64	NI	Personal computer
SPS 34	260	NI	NI
SPS 35	2000	3	Personal computer
SPS 36	2500	11	Personal computer
SPS 37	200	NI	Personal computer
SPS 38	100	NI	Personal computer
SPS 39	256	152.71	NI
SPS 40	250	4.5	Embedded System
SPS 41	150	12.8	Embedded System
SPS 42	200	46.4	Personal computer
SPS 43	200	5	Embedded System
SPS 44	NI	233.4	NI
SPS 45	200	NI	NI
SPS 46	250	NI	NI
SPS 47	NI	NI	NI
SPS 48	200	NI	Embedded System
SPS 49	800	NI	NI
SPS 50	400	NI	Embedded System
SPS 51	500	NI	Personal computer
SPS 52	240	NI	Personal computer
SPS 53	32	NI	Personal computer
SPS 54	NI	190	NI
SPS 55	300	NI	NI
SPS 56	400	227.76	NI
SPS 57	160	<16	NI
SPS 58	160	<16	NI
SPS 59	200	2	NI
SPS 60	200	50	Personal computer
SPS 61	200	<50	NI
SPS 62	167	6	Personal computer
SPS 63	250	<50	NI
SPS 64	250	<50	NI
SPS 65	200	<50	Personal computer

**NI**: Not indicated.

**Table 11:** The number of gestures recognized (i.e., classes), number of gestures per person in the training set (NGpPT), the number of people who participated in the training (NPT), the number of gestures per person in the evaluation set (NGpPE), the type of gestures recognized, the state of the EMG data used, and the duration of the gestures (DG).

ID SPS	Classes	NGpPT	NPT	NGpPE	TGR	StEMG	DG (s)
SPS 1	4	20	13	20	Static	NI	5
SPS 2	5	25	10	150	Static	NI	STG
SPS 3	6	300	1	300	Static	Steady and Transient	4
SPS 4	8 *	NI	NI	NI	Static	NI	NI
SPS 5	9 *	90	5	NI	Static	NI	NI
SPS 6	13 *	65	8	65	Static	NI	4
SPS 7	5	25	14	25	Static	NI	NI
SPS 8	5	25	10	150	Static	NI	STG
SPS 9	5	25	10	150	Static	NI	STG
SPS 10	3 *	15	1	NI	Static	NI	NI
SPS 11	8 *	80	6	NI	Static	NI	10
SPS 12	10	NI	NI	NI	Static	NI	NI
SPS 13	9	NI	17	NI	Static	NI	NI
SPS 14	4	NI	NI	NI	Static	NI	1
SPS 15	3	18	3	150	Static	NI	NI
SPS 16	10	300	4	NI	Static	Transient	STG
SPS 17	17 *	13600	5	1700	Static	NI	NI
SPS 18	20	NI	NI	NI	Static	NI	30
SPS 19	6	NI	NI	NI	Static	NI	STG
SPS 20	7 *	21	NI	NI	Static	NI	1
SPS 21	13 *	65	8	65	Static	NI	4-6
SPS 22	4	NI	20	NI	Static	NI	NI
SPS 23	6	NI	80	NI	Static	NI	NI
SPS 24	6	300	1	300	Static	NI	NI
SPS 25	26	1040	1	520	Static and Dynamic	NI	2
SPS 26	3	NI	4	NI	Static	NI	NI
SPS 27	7 *	NI	4	NI	Static	NI	NI
SPS 28	6 *	18	9	42	Static	Steady	3
SPS 29	4	NI	NI	NI	Static	NI	NI
SPS 30	3 *	NI	1	NI	Static	NI	NI
SPS 31	6 *	54	5	36	Static	NI	5-6
SPS 32	8	NI	10	NI	Static	NI	5
SPS 33	10	500	6	1800	Static	NI	STG
SPS 34	7 *	28	19	84	Static	NI	0.95
SPS 35	5	250	50	250	Static	NI	STG
SPS 36	6 *	30	10	150	Static	NI	STG
SPS 37	5 *	20	6	10	Static	NI	5
SPS 38	2 *	NI	5	NI	Static	NI	NI
SPS 39	5	40	5	160	Static	NI	4
SPS 40	9 *	90	10	90	Static	Steady	5
SPS 41	9 *	540	3	540	Static	Steady	3
SPS 42	6	NI	8	NI	Static	NI	5
SPS 43	8	NI	NI	NI	Static	Steady and Transient	5
SPS 44	6 *	180	1	150	Static	NI	NI
SPS 45	47	94	5	47	NI	Steady	6
SPS 46	7 *	NI	17	NI	Static	NI	20
SPS 47	9	NI	1	NI	Static	NI	10
SPS 48	6 *	NI	4	150	Static	Transient	STG
SPS 49	4	40	7	100	Static	NI	NI
SPS 50	5	510	NI	NI	Static	NI	NI
SPS 51	8 *	528	1	176	Static	NI	2
SPS 52	7 *	56	6	48	Static	NI	5
SPS 53	9	450	20	450	Static	NI	1
SPS 54	9 *	250	NI	60	Static	NI	5
SPS 55	13	NI	10	NI	Static	Steady	6
SPS 56	5	25	12	150	Static	NI	2 (training), and 5 (testing)
SPS 57	5 *	10	9	48	Static	NI	3
SPS 58	7 *	28	10	144	Static	NI	2
SPS 59	14	56	10	84	Static	NI	7
SPS 60	11 *	33	10	6	Static	NI	3
SPS 61	5 *	75	10	72	Static	Steady	4
SPS 62	9 *	32	10	48	Static	NI	12
SPS 63	8	32	4	40	Static	NI	3
SPS 64	5 *	40	11	270	Static	ni	3
SPS 65	7 *	21	11	48	Static	Steady and Transient	3

**NI**: Not indicated; *: Including the rest gesture; **NGpPT**: Number of Gestures per Person in the Training set; **NPT**: Number of People Who Participated in the Evaluation; **NGpPE**: Number of Gestures per Person in the Evaluation set; **TGR**: Type of Gestures Recognized; **StEMG**: State of the EMG; **DG**: Duration of the Gestures; **STG**: Short-Term Gesture.

**Table 12:** The evaluation metrics for machine learning used by the 56 HGR models.

Evaluation Metric	IDs of the SPS
Accuracy	All HGR models, except SPS 18, SPS 37, and SPS 38
Recall	SPS 2, SPS 3, SPS 4, SPS 8, SPS 9, SPS 12, SPS 14, SPS 17, SPS 18, SPS 19, SPS 24, SPS 26, SPS 28, SPS 29, SPS 31, SPS 33, SPS 35, SPS 36, SPS 39, SPS 40, SPS 42, SPS 44, SPS 46, SPS 49, SPS 53, SPS 55, and SPS 56
Precision	SPS 2, SPS 8, SPS 9, SPS 14, SPS 35, SPS 36, SPS 44, SPS 53, and SPS 56
Accuracy per User	SPS 1, SPS 5, SPS 6, SPS 16, SPS 26, SPS 31, SPS 33, SPS 38, SPS 39, SPS 48, SPS 52, SPS 53, and SPS 56
Recall per User	SPS 15, and SPS 26
Precision per User	SPS 15, and SPS 39
Median of the Accuracy per User	SPS 6
Standard Deviation of the Accuracy per User	SPS 1, SPS 5, SPS 7, SPS 20, SPS 35
Standard Deviation of the Accuracy per Class	SPS 17
Standard Deviation of each User Accuracy	SPS 5
Standard Deviation of the Recalls of each Class	SPS 17
Kappa Index	SPS 46
Accuracy Error	SPS 37

**Table 13:** The accuracy, number of people who participated in the evaluation, type of data set (i.e., balanced or unbalanced), and the use of cross-validation by the 56 HGR models.

ID SPS	Model Classification Accuracy (%)	NPE	Type of Data Set	Cross-Validation
SPS 1	94.00	13	balanced	NI
SPS 2	90.70	10	balanced	NI
SPS 3	99.00	1	balanced	yes
SPS 4	93.00	10	balanced	NI
SPS 5	92.20	5	balanced	yes
SPS 6	82.39	8	unbalanced	NI
SPS 7	95.64	14	balanced	yes
SPS 8	86.00	10	balanced	NI
SPS 9	89.50	10	balanced	NI
SPS 10	85.00	1	NI	NI
SPS 11	97.35	6	NI	NI
SPS 12	89.00	NI	balanced	NI
SPS 13	82.43	17	NI	NI
SPS 14	87.00	NI	NI	NI
SPS 15	90.00	3	balanced	NI
SPS 16	94.00	4	balanced	yes
SPS 17	89.38	5	balanced	NI
SPS 18	NI	NI	NI	yes
SPS 19	97.30	NI	NI	NI
SPS 20	97.90	18	NI	NI
SPS 21	89.00	8	balanced	yes
SPS 22	97.50	20	NI	NI
SPS 23	97.50	80	balanced	yes
SPS 24	71.00	1	balanced	NI
SPS 25	82.30	1	balanced	yes
SPS 26	93.25	4	balanced	NI
SPS 27	89.20	4	NI	NI
SPS 28	91.80	9	balanced	yes
SPS 29	93.00	10	balanced	NI
SPS 30	83.90	1	NI	yes
SPS 31	88.00	5	balanced	yes
SPS 32	95.00	10	balanced	yes
SPS 33	90.00	6	balanced	yes
SPS 34	98.31	17	balanced	yes
SPS 35	85.08	60	balanced	NI
SPS 36	90.1	10	balanced	NI
SPS 37	NI	6	balanced	NI
SPS 38	NI	5	NI	NI
SPS 39	96.08	5	balanced	yes
SPS 40	99.03	10	balanced	NI
SPS 41	97.01	3	balanced	yes
SPS 42	91.93	8	balanced	NI
SPS 43	98.15	NI	balanced	NI
SPS 44	96.70	1	balanced	NI
SPS 45	82.11	5	NI	yes
SPS 46	99.78	17	balanced	NI
SPS 47	90.30	1	balanced	yes
SPS 48	94.14	4	balanced	NI
SPS 49	90.00	7	balanced	NI
SPS 50	73.00	NI	NI	NI
SPS 51	95.31*	1	balanced	NI
SPS 52	95.20	6	balanced	yes
SPS 53	95.00	20	balanced	NI
SPS 54	95.10	NI	NI	NI
SPS 55	99.20	10	NI	NI
SPS 56	98.70	12	balanced	NI

**NI**: Not indicated; **NPE**: Number of people who participated in the Evaluation; *: This is recognition accuracy (i.e., this model determines what gesture and when this gesture was performed by a person); **yes**: This model uses cross-validation.

**Table 14:** Metrics of the target achievement test used by the nine HGR models.

Metric	Description
Throughput	Ratio between the index of difficulty and the movement time, which is the time (in seconds) [107].
Path Efficiency	Ratio between the straight line distance and the actual distance traveled [107,126].
Overshoot	Ratio between overshoots and number of targets. The ability to stop on a target [107,126].
Average Speed	Average nonzero speed of the cursor over the course of the trial [107,126].
Completion Rate	Ratio between the completed trials and the number of trials within the allowed time (i.e., trial time) [50,126].
Stopping Distance	Total distance traveled (path length) during the dwell time [108].
Completion Time	Time from movement initiation to the completion of the trial [31].
Real-time Accuracy	Ratio between correct predictions and number of predictions during the completion time [127].
Length Error	Ratio between distance beyond the total required distance, and the total required distance [31].
Reaction Time	Time from a target appearance and the first move of the cursor/virtual prosthesis [113].

## Abbreviations

The following abbreviations are used in this manuscript:

CL	Classification
DA	Data Acquisition
DAT	Data Analysis Time
DCT	Data Collection Time
DG	Duration of the Gestures
EMG	Surface Electromyography
FE	Feature Extraction
FLT	Fitts’ Law Test
HCI	Human–Computer Interaction
HGR	Hand Gesture recognition
ID	Index of Difficulty
IMU	Inertial Measurement Unit
ML	Machine Learning
MMDC	Mathematical Model for a Dynamic Contraction
MMSC	Mathematical Model for a Static Contraction
MT	Movement Time
MU	Motor Unit
MUAP	Motor Unit Action Potential
NGpPE	Number of Gesture per Person in the Evaluation Set
NGpPT	Number of Gestures per Person in the Training Set
NPT	Number of People who Participated in the Training
POSTP	Postprocessing
PREP	Preprocessing
SEGM	Segmentation
SLR	Systematic Literature Review
SPS	Selected Primary Study
TAC	Target Achievement Control Test
